# *Leishmania* species and clinical characteristics of Pacific and Amazon cutaneous leishmaniasis in Ecuador and determinants of health-seeking delay: a cross-sectional study

**DOI:** 10.1186/s12879-023-08377-8

**Published:** 2023-06-12

**Authors:** Jacob M. Bezemer, Byron P. Freire-Paspuel, Henk D. F. H. Schallig, Henry J.C. de Vries, Manuel Calvopiña

**Affiliations:** 1 Fundación Misión Cristiana de Salud, Hospital Shell, Shell, Pastaza, Ecuador; 2 Department of Medical Microbiology and Infection Prevention, Laboratory for Experimental Parasitology, Amsterdam UMC location University of Amsterdam, Amsterdam, the Netherlands; 3 Amsterdam Institute for infection and Immunity (AII), Infectious Diseases Program, Amsterdam, the Netherlands; 4 Laboratorios de Investigación, Universidad de las Américas, Quito, Ecuador; 5 Hospital Universitari Vall d’Hebron, Vall d’Hebron Research Institute, Barcelona, Spain; 6 Department of dermatology, Amsterdam UMC location University of Amsterdam, Amsterdam, the Netherlands; 7 Public Health Service, Center for Sexual Health, Department of Infectious Diseases, Amsterdam, the Netherlands; 8 Facultad de Medicina, Universidad de las Américas, OneHealth Research Group, Quito, Ecuador

**Keywords:** Leishmaniasis, cutaneous, Leishmaniasis, epidemiology, Phylogeny, Time-to-treatment, Ecuador

## Abstract

**Background:**

Cutaneous Leishmaniasis (CL) affects up to 5.000 people in Ecuador each year. *L. guyanensis* and *L. braziliensis* are the most common of the eight CL-causing *Leishmania* species. Earlier CL research concentrated on the easily accessible Pacific region. This study aims to describe the *Leishmania* species in Pacific and Amazon ecoregions, to analyze regional differences in CL patient clinical presentation, and to identify determinants of health-seeking delay.

**Methods:**

All cases in this cross-sectional study were diagnosed using smear slide microscopy, PCR, or both. Cytochrome B gene sequencing was used to identify the causative *Leishmania* species in qPCR-positive samples.

**Results:**

This study included 245 patients, with 154 (63%) infected in the Pacific region and 91 (37%) infected in the Amazon. Causative *Leishmania* species were identified in 135 patients (73% of qPCR positives). *L. guyanensis* was identified in 76% (102/135) of the samples and *L. braziliensis* in 19% (26/135). The Pacific region had a low prevalence of 6% (5/89) of *L. braziliensis*. For the first time, we report *L. guyanensis* from the central Amazon, *L. braziliensis* from the northern Pacific, and *L. lainsoni* from both the central Amazon and northern Pacific. Amazon cases had a longer median health-seeking delay in months (2.0, IQR 3.0) than Pacific cases (1.0, IQR 1.5). Prolonged health-seeking delay was associated with older age, Amerindian ethnicity, infection at lower altitudes, non-ulcerative lesions, and lesions on the lower limbs.

**Conclusions:**

In the Pacific region, health-seeking delay is relatively short and *L. braziliensis* prevalence remains low. Limited access to health care and stigma might explain the prolonged health-seeking delay in the Amazon. We recommend larger studies on the distribution of *Leishmania* species in Amazon CL cases and additional regional research into diagnostic test accuracy. Furthermore, the determinants of health-seeking delay in Ecuador should be investigated further.

**Supplementary Information:**

The online version contains supplementary material available at 10.1186/s12879-023-08377-8.

## Introduction

### Background

**Fig. 1:**
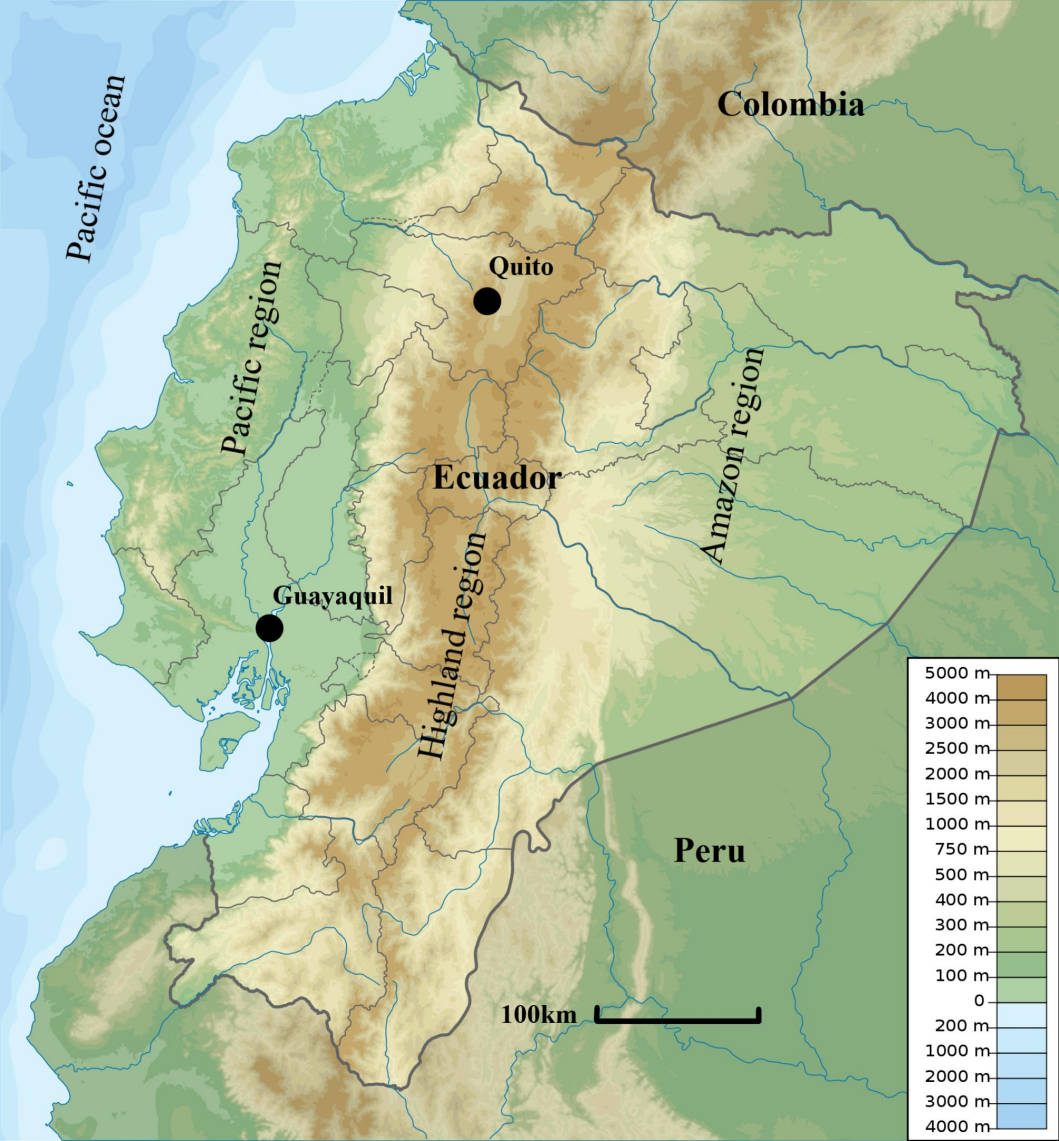
Altitude map of Ecuador with the coastal Pacific, Highland, and Amazon regions. The image is adapted from Wikipedia by the authors and is available under the Creative Commons CC0 1.0 Universal Public Domain Dedication [9].

Leishmaniasis is a vector-transmitted parasitic disease that leads to visceral (VL), cutaneous (CL), or mucosal (ML) lesions [1]. CL could cause ulcers and nodular lesions of the skin that heal spontaneously with scarring over months to years. VL affects the reticuloendothelial system and is lethal if left untreated. ML ulcerates and deforms mucous membranes, does not heal spontaneously, and may be lethal [2]. The World Health Organization (WHO) classifies Leishmaniasis as a Neglected Tropical Disease (NTD) because it disproportionally affects poor and vulnerable populations, lacks funding, and requires research to improve understanding, diagnosis, treatment, and prevention (e.g. vaccines and vector control) of the disease [3, 4]. Twenty-two *Leishmania* species are pathogenic to humans with each primarily causing one or two of the disease manifestations CL, VL, or ML [5]. Their distribution is limited geographically depending on the interaction between the parasite, animal reservoir, and sand fly vector (*Phlebotomus* and *Lutzomyia*) [2, 6]. Every year, leishmaniasis affects 700.000 to 1 million people worldwide including an estimated 58.000 in South America of which 5.000 in Ecuador [7, 8]. The Andean Mountain range divides the Ecuadorian mainland into three regions: The Pacific on the west, the Highlands in the middle, and the Amazon on the east (Fig. 1).

No VL cases have been reported in Ecuador [10]. CL is endemic (1.7–46.4/1.000 inhabitants yearly) in 12 cantons in the Pacific region with a total surface of 11.000 Km^2^ and an estimated 749.000 inhabitants but ML is uncommon. Leishmaniasis is not endemic in the Highland region. In the Amazon, CL, and ML are endemic (1.7–21.2/1.000 inhabitants yearly) in all the cantons with a total surface of 120.000 Km^2^ and an estimated 957.000 inhabitants [8, 11–13]. *Leishmania guyanensis* and *L. braziliensis* are dominant among eight recorded species. *L. guyanensis* is only found in CL patients but *L. braziliensis* in both CL and ML patients. Studies on *Leishmania* species in Ecuador have primarily focused on the accessible Pacific region and included only a few patients from the remote Amazon [14, 15]. They reported a predominance of *L. guyanensis* in the Pacific and *L. braziliensis* in the Amazon. Kato et al. reported an increase in *L. braziliensis* cases in the Pacific in 2016, possibly increasing the risk of ML [14]. Because this study was not followed up on, the need for active surveillance of ML cases remains unclear. In Ecuador, the reservoirs and vectors of *L. guyanensis* and *L. braziliensis* are unknown.

Most of the Pacific region’s population belongs to the Mestizo group (descendants from Europeans mixed with Amerindians), with intermediate levels of poverty, relatively low rates of analphabetism, and relatively high accessibility via public transportation. The Amazon population auto-identifies for almost 50% as Amerindian, has high levels of poverty, high rates of analphabetism, and low accessibility via public transportation [16–18]. The Amerindian population is marginalized and lives partially in the lowland rainforest with only air travel access [19–21]. Regional differences in *Leishmania* species and human population characteristics might influence clinical characteristics (e.g., age, gender, lesion location, lesion types, health-seeking delay), and response to treatment [22, 23]. Nevertheless, no studies have compared the presentation of CL cases in the Pacific and Amazon regions hampering surveillance, prevention, timely diagnosis, and subsequent treatment.

### Objectives

The objectives of this paper are to describe the *Leishmania* species in Pacific and Amazon ecoregions, to analyze regional differences in CL patient clinical presentation, and to identify determinants of health-seeking delay.

## Methods

### Participants

Patients were included in private and public primary health care centers and hospitals from the subtropical Pacific region of the Pichincha province and the Amazon Napo, Pastaza, and Morona Santiago provinces. Patients were included from January 2019 through June 2021. All participants or their legal representatives provided written informed consent. The research protocol was approved by the ethical committee of the `Universidad Internacional del Ecuador’ registration number: UIDE-FCM-EDM-COM-18-0069, and the Ecuadorian Ministry of Health, registration number: MSPCURI000284-3, prior to its initiation. All the methods were carried out in agreement with the guidelines of the Ministry of Health of Ecuador and in accordance with the declaration of Helsinki. The participating centers (public and private) provided free leishmaniasis care including consultation, laboratory testing, and treatment (intramuscular meglumine antimoniate) in accordance with the national protocol of the Ecuadorian Ministry of Health [24].

Cases were recruited for the current study if they matched the following inclusion criteria: one or more CL-suspected skin lesions, age range 0–90, and signed informed permission. All patients (regardless of ethnicity, place of residence, or pregnancy status) sent to the collaborating facilities for a microscopic smear slide evaluation of a skin lesion suggestive of CL were eligible. Patients were included by the doctor, nurse, or laboratory technician during normal workflow before lesion sampling. Patients were excluded from the study if they did not meet the inclusion criteria or the *Leishmania* infection could not be confirmed either by Polymerase Chain Reaction (PCR) or microscopy.

### Laboratory tests

An experienced laboratory technician took a skin scraping from the inner border of the lesions to perform a smear slide. In the participating health centers, smear slides were Giemsa stained according to the protocol of the Ecuadorian Ministry of Health and read with light microscopy [25, 26]. The result of microscopy was reported as “positive” when *Leishmania* amastigotes were identified. A quality check was performed on all positive samples. Immediately after the skin scraping for smear slide, a filter paper, 903 Protein Saver Card (Whatman, Newton Center, MA), was pressed three times on the scraping site and dried at room temperature. Filter papers were processed at the `Universidad de las Americas’ research laboratory in Quito, Ecuador. DNA was extracted from a 2 × 2mm piece that was cut from each filter paper and transferred to a 1.5mL tube containing 200µL of 10% (wt/vol) Chelex 100 (Sigma-Aldrich, USA) [27] and 10µL of Proteinase K (Invitrogen, USA). Samples were strongly vortexed for 5 min and subsequently incubated at 56°C for 60 min and 96°C for 20 min in an Eppendorf ThermoMixer C. The supernatant containing the DNA was separated from the Chelex resin and transferred to a new 1.5 mL tube. The presence of *Leishmania* DNA in the extracted samples was evaluated by probe-based real-time PCR (qPCR) following the protocol described by Bezemer et al. [28]. Identification of samples that showed amplification of *Leishmania* 18S rDNA by qPCR was subsequently performed by nested PCR and sequencing of the cytochrome B (*Cyt* B). The first PCR reaction, using outer primers L.cyt-AS (5’-GCG GAG AGR ARG AAA AGG C-3’) and L.cyt-AR (5’-CCA CTC ATA AAT ATA CTA TA-3’), was prepared in a total volume of 15µL containing: 0.06U/µL of Platinum Taq DNA Polymerase (Invitrogen, USA), 0.5µM of each primer, 2.5mM of MgCl_2_, 0.3mM of dNTP mix, and 2µL of DNA sample. The following thermal cycler protocol was used: 1 cycle at 94 °C for 2 min; 35 cycles at 95 °C for 30 s, 55 °C for 30 s, 72 °C for 1 min; 1 cycle at 72 °C for 5 min. Then, 1µL of the product of the first PCR was reamplified in the second PCR using primers L.cyt-S (5’-GGT GTA GGT TTT AGT YTA GG-3’) and L.cyt-R (5’-CTA CAA TAA ACA AAT CAT AAT ATR CAA TT-3’) following the same protocol [14]. Differentiation between *L. braziliensis* and *L. peruviana* was performed by sequencing the Mannose Phosphate Isomerase (MPI) gene using MPI-S (5’-GCT CTT CCT GTC GGA CAG CGA GC-3’) and MPI-R (5’-TCA CTC TCG AAG GGA GTT CG-3’) primers [29, 30]. The sequencing reaction was prepared using BigDye Terminator v3.1 Cycle Sequencing Kit (Applied Biosystems, USA) and capillary electrophoresis were run in a 3500 Genetic Analyzer (Applied Biosystems, USA). The resulting sequences were compared with the NCBI database by applying the Basic Local Alignment for species determination [31]. Additionally, a phylogenetic tree was obtained using the Geneious R11 software and the Tamura-Nei model. The following species’ reference sequences described by Kato et al. [29] were included in the tree: *L. braziliensis* (GenBank accession number AB095966), *L. guyanensis* (AB095969), *L. lainsoni* (AB433280*), L. naiffi* (AB433279), *L. panamensis* (AB095968), *L. shawi* (AB433281) and *L. peruviana* (AB433282) [32]. In addition to the qPCR, an endpoint PCR was performed that differentiated between the *Viannia* and *Leishmania* subgenus, having a sensitivity of only 50% as published elsewhere [33]. The endpoint PCR was discontinued after the first 100 samples due to human resources problems as a result of the COVID-19 pandemic.

### Study variables

The following demographic data of study participants were recorded: Age in years, gender (male or female), ethnicity as recognized by the Ecuadorian government [34], presumed place of infection, health-seeking delay in months (defined as the time since onset of symptoms to inclusion in the study and based on recall), lesion type (ulcer, nodular, or other), number of skin lesions, and the location of lesion(s) (indicated with pencil on a person image by the health professional). GPS coordinates of the presumed place of infection were estimated with Google Maps [35]. Altitude in meters was defined as the altitude of the airstrip as reported by the general directorate of civil aviation for hinterland villages [35]. Altitudes of other places were estimated with topographic-map.com [36]. We compared the clinical characteristics of CL patients and *Leishmania* species prevalence between the Pacific and Amazon regions as these regions are geographically separated. Patient ethnicity was grouped into Mestizo and Amazon Amerindian to allow analysis. Lesion location was categorized into four major groups for analysis: Head, Trunk, Upper limbs, and Lower limbs. The sample size was a convenience sample calculated using the average annual number of CL tests performed by the participating centers in the five years preceding the start of the study. With a 24-months inclusion period, the sample size was 600 (50% of the expected cases). When the first nationwide COVID-19 quarantine started, the number of inclusions fell dramatically as patients feared to visit healthcare centers, leaving lesions unattended that may have been cured spontaneously, or to give informed consent. The research team had funding and personnel to prolong the inclusion period from 24 months to 29 months until obtaining more than half of the expected number of inclusions and had to stop then.

### Analysis

Two independent investigators entered collected data into an electronic data capture system and the data were computer validated [37]. Allocation to the Pacific or Amazon group was based on the infection region. All calculations were done in SPSS Statistics version 28, considering P < 0.05 as statistically significant [38]. To address the second objective, associations of CL patient clinical characteristics with the region of infection were tested with the independent samples T-test in normally distributed continuous variables, the proportions (Wald) or Fishers exact (when proportions < 1) test in dichotomous or categorical variables, and the Mann-Whitney U test in non-parametric continuous variables. The patient was excluded from a particular comparison if data were missing for that comparison. The continuous variable ‘Health-seeking delay’ was transformed into a binary variable ‘Health-seeking delay ≥ one month’ for analysis of its determinants. The one-month cut was chosen because bacterial lesions, which are the most common differential diagnosis, mostly heal in two weeks, leaving 14 days for early health-seeking. To address the third objective, associations of CL patient clinical characteristics with ‘Health-seeking delay ≥ one month’ (prolonged health-seeking delay) were tested as for region of infection.

### Confounders of prolonged health-seeking delay in the Amazon

Blockwise addition of the variable ‘Infected in the Amazon’ to a multivariable binary logistic regression model of ‘Health-seeking delay ≥ one month’ was used to test if non-included confounders in the Amazon influence prolonged health-seeking delay. Therefore, the following determinants of health-seeking delay were included in Block 1 of a multivariable binary logistic regression model: Age (years), Male gender, Amerindian ethnicity, Altitude of the place of infection (hectometers), Number of lesions, and Lesion location head and neck. Age was included because it influences illness knowledge and attitudes [39, 40]. Gender because it may influence the moment of health-seeking as well as the decision-making process [41]. Amerindian ethnicity because cultural values and practices as well as language barriers may influence the moment of health-seeking [42]. Altitude because it determines the distance to referral centers [17]. Number of lesions because they may increase the perceived severity. Lesion location head and neck because there it’s difficult to hide lesions and differences might indicate stigma [43]. The variable ‘Infected in the Amazon’ was added to Block 2. The Omnibus Tests of Model Coefficients was applied to assess if ‘infected in the Amazon’ improved this model indicating that non-included confounders in that region influence health-seeking delay. Lesion types (ulcer, nodular, or other) were not included in this model because of sparse results in at least one cell in the cross-tabulations. This article was written by the STROBE checklist for cross-sectional studies [44].

## Results

**Fig. 2:**
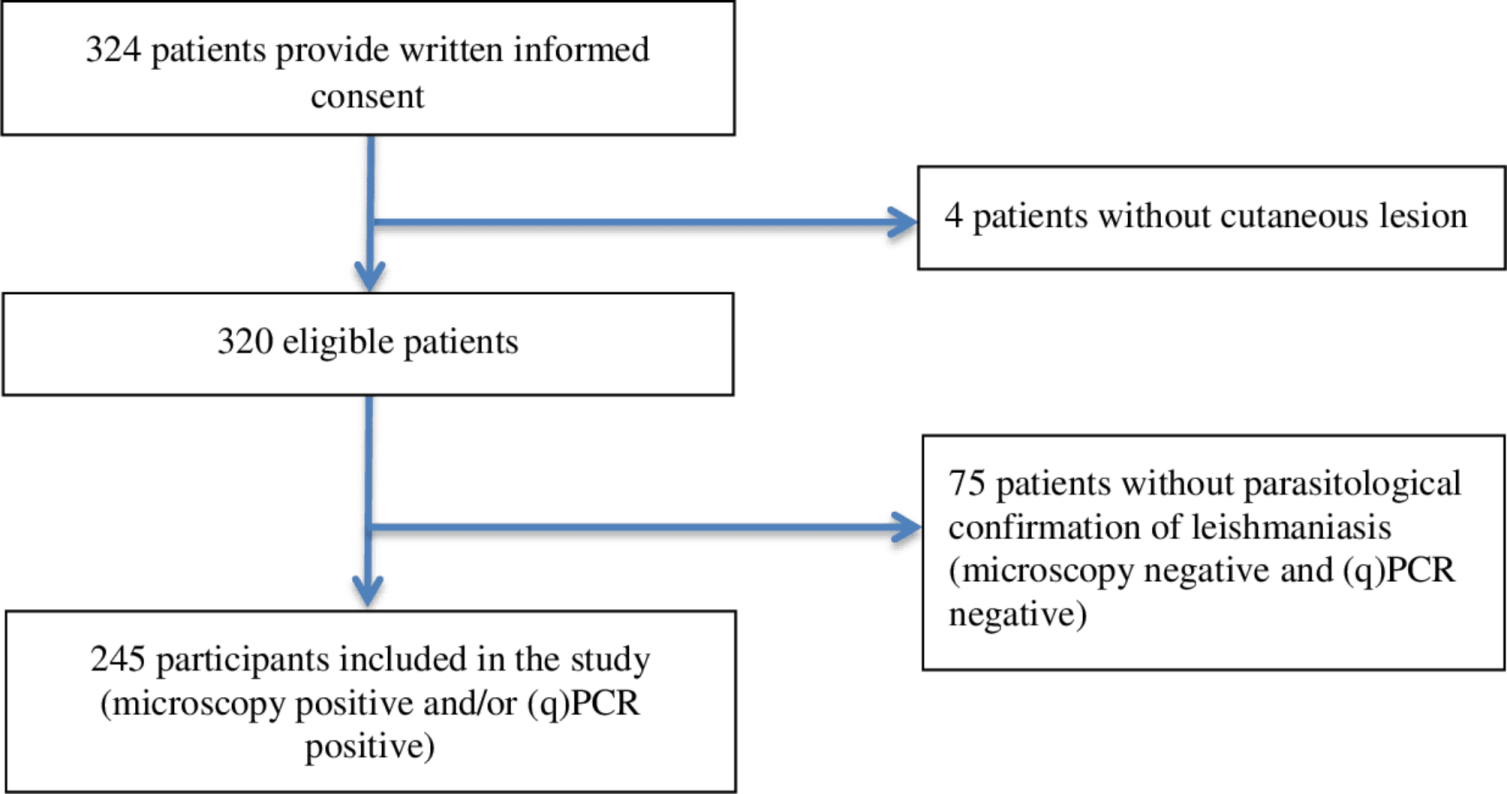
Flow diagram of participants

The presence of *Leishmania* parasites was confirmed with PCR and/or microscopy in 245 patients who were included in this study (Fig. 2). 179 (73%) patients were included by a public and 66 (27%) by a private healthcare center.

**Fig. 3:**
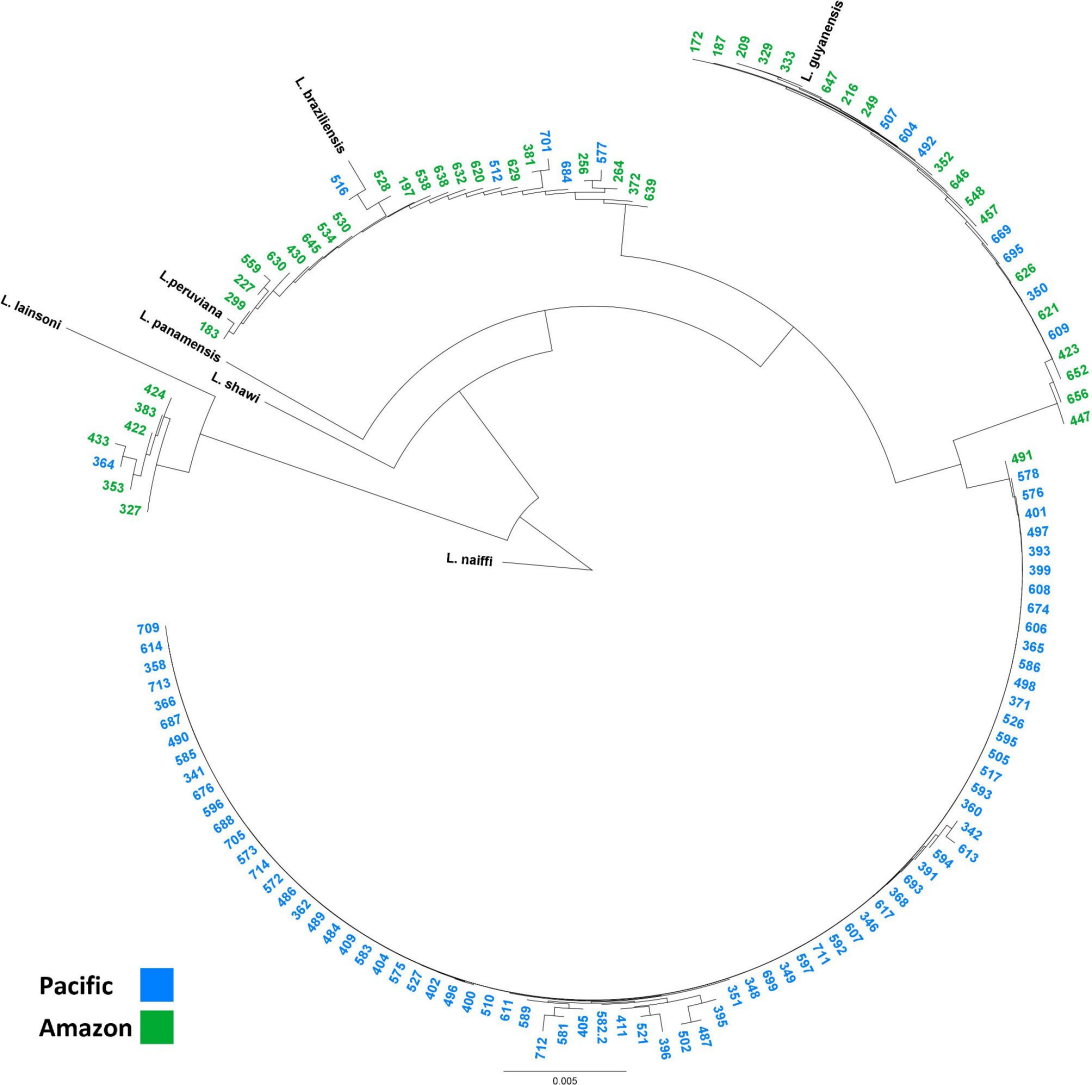
Phylogenetic tree of a Cytochrome B gene fragment from 135 Ecuadorian CL patient samples

Infecting *Leishmania* species could be determined in 135 (73%) of the qPCR-positive samples. PCR of Cyt B did not amplify in the remaining samples and sequencing was impossible. In the Pacific region, Pichincha accounted for 93% of the cases, of which 94% were caused by *L. guyanensis*. In the Amazon region, there was a similar distribution of cases between *L. braziliensis* (41%) and *L. guyanensis* (46%). In the Amazon region, the province of Pastaza accounted for 72% of the cases. All *L. braziliensis* samples belonged to a single clade in the phylogenetic tree of the Cyt B sequences that included *L. peruviana*. The *L. guyanensis* samples were divided into a subclade with 76 (99%) Pacific samples and a subclade with 18 (72%) Amazon samples (see Table 1, Additional files 1 and 2, Fig. 3, and sequences submitted to GenBank with accession numbers: OQ608467-OQ608601).

**Table 1:** Clinical characteristics and species determination in 245 cutaneous leishmaniasis patients from the Pacific and Amazon regions

Patient characteristic (N missing for variable)	Pacific	Amazon	Two-sided P^a^	Total
Number of Patients (%)	154 (63)	91 (37)		245 (100)
**General characteristics**				
Mean age in years (SD)	22.4 (17.4)	30.8 (20.4)	< 0.01^b^	25.5 (19.0)
Males (%)	83 (54)	57 (63)	0.18	140 (57)
Mestizo (%)	154 (100)	25 (27)		179 (73)
Amazon Amerindian (%)	0 (0)	66 (73)	< 0.01b	66 (27)
Median altitude of place of infection in hectometers (IQR)	5.5 (5.0)	3.8 (3.2)	0.03^b^	4.6 (4.6)
**Clinical presentation (1)**				
Median health-seeking delay in months (IQR)	1.0 (1.5)	2.0 (3.0)	< 0.01^b^	1.0 (1.5)
Lesion type: ulcer (%)	143 (93)	87 (96)	0.39	230 (94)
Median number of lesions (IQR)	1 (1)	1 (0)	0.48	1 (1)
**Body location of the lesion** ^**c**^				
Head and neck (%)	45 (27)	10 (10)	< 0.01^b^	55 (21)
Trunk (%)	18 (11)	15 (15)	0.31	33 (12)
Upper limbs (%)	67 (39)	43 (43)	0.57	110 (41)
Lower limbs (%)	39 (23)	31 (32)	0.15	70 (26)
***Leishmania*** **species (110)**				
*L. guyanensis*	83 (93)	19 (41)	< 0.01^b^	102 (76)
*L. braziliensis*	5 (6)	21 (46)	< 0.01^b^	26 (19)
*L. lainsoni*	1 (1)	6 (13)	< 0.01^b^	7 (5)

N = Number, P = Probability, SD = Standard Deviation, IQR = Interquartile Range

^a^ Comparing Pacific to Amazon with the Independent samples T, Wald, Fishers exact, or Mann-Whitney U test

^b^ Statistically significant

^c^ Patients with lesions on different body regions were counted more than once

The MPI gene could be sequenced in 18/26 (69%) *L. braziliensis* samples and comparison of the resulting sequences excluded the presence of *L. peruviana* (see Additional file 3 and sequences submitted to GenBank with accession numbers OQ608603-OQ608620 [32]).

### Determinants of health-seeking delay

The duration of health-seeking delay was known for 244 of 245 confirmed patients. Patients with health-seeking delay ≥ one month were significantly older (27.7 compared to 21.9), more often had Amerindian ethnicity (34% compared to 14%), were infected in the Amazon (45% compared to 14%), presented with no ulcer (92% compared to 98%), were less often infected on the head or neck (15% compared to 32%) and more often on the lower limbs (32% compared to 15%) (Table 2). The multivariable binary logistic model of ‘health-seeking delay ≥ one month’ did not improve significantly after the addition of the determinant ‘Infected in the Amazon’ to ‘age’, ‘gender’, ‘Amerindian ethnicity’, ‘altitude of place of infection’, and ‘lesion on the head or neck’ (Table 3).

**Table 2:** Patient characteristics and prolonged health-seeking delay (≥ one month) in 244 confirmed cutaneous leishmaniasis patients in Ecuador

Patient characteristic (N missing for variable)	Health-seeking delay < 1 month	Health-seeking delay ≥ 1 month	Two-sided P^a^	All patients
Number of Patients (%)	88 (36)	156 (64)		244 (100)
**General characteristics**				
Mean age in years (SD)	21.9 (17.1)	27.7 (19.7)	0.02^b^	25.6 (18.9)
Males (%)	52 (59)	87 (56)	0.62	139 (57)
Mestizo (%)	76 (86)	103 (66)		179 (73)
Amazon Amerindian (%)	12 (14)	53 (34)	< 0.01^b^	65 (27)
**Characteristics of the area of infection**				
Pacific region (%)	69 (78)	85 (55)		154 (63)
Amazon region (%)	19 (22)	71 (45)	< 0.01^b^	90 (37)
Median altitude of place of infection in hectometers (IQR)	6.3 (6.0)	3.7 (3.5)	< 0.01^b^	4.7 (4.6)
**Clinical presentation**				
Lesion type: ulcer (%)	87 (98)	142 (92)	0.01^b^	229 (93)
Lesion type: nodule (%)	2 (2)	7 (4)	0.50	9 (4)
Lesion type: other (%)	0 (0)	7 (4)	0.05	7 (3)
Median number of lesions (IQR)	1 (0)	1 (1)	0.11	1 (1)
**Body location of the lesion** ^**c**^				
Head and neck (%)	30 (32)	25 (15)	< 0.01^b^	55 (21)
Trunk (%)	14 (15)	18 (10)	0.33	32 (12)
Upper limbs (%)	36 (38)	74 (43)	0.33	110 (41)
Lower limbs (%)	14 (15)	56 (32)	< 0.01^b^	70 (26)

N = Number, P = Probability, SD = Standard Deviation, IQR = Interquartile Range

^a^ Comparing Health-seeking delay < 1 month and ≥ 1 month with the independent samples T, Wald, Fishers exact, or Mann-Whitney U test

^b^ Statistically significant

^c^ Patients with lesions on different body regions were counted more than once

**Table 3:** Blockwise addition of the variable ‘infected in the Amazon´ to a multivariable model of health-seeking delay ≥ one month in 244 confirmed cutaneous leishmaniasis patients in Ecuador

	Block 1	Block 2		P^a^
Overall significance	< 0.01	< 0.01		
Nagelkerke R^2^	27.3%	28.1%		
Percentage correctly classified	63.9%	74.6%		
**Variable**	**Odds ratios (95% CI)**		**Odds ratios (95% CI)**	
Age in years	1.02 (1.00-1.03)		1.01 (1.00-1.03)	
Male gender	0.83 (0.46–1.51)		0.80 (0.44–1.47)	
Amerindian ethnicity	1.85 (0.87–3.92)		1.00 (0.30–3.32)	
Altitude of place of infection in hectometers	0.77 (0.69–0.85)		0.76 (0.69–0.85)	
Number of lesions	1.26 (0.94–1.68)		1.26 (0.93–1.70)	
Lesion location: Head and neck	0.44 (0.22–0.90)		0.47 (0.23–0.95)	
Infected in the Amazon			2.04 (0.70–5.92)	0.18

CI = Confidence Interval, NA = Not Applicable

^a^Omnibus Tests of Model Coefficients comparing Block 1 to Block 2

## Discussion

This study describes the infecting *Leishmania* species in 135 Ecuadorian CL cases. Additionally, the clinical presentation and determinants of health-seeking delay of over 240 confirmed CL cases were compared between the Pacific and Amazon regions. For the first time, we report *L. guyanensis* in the provinces of Napo, Pastaza, and Morona Santiago, *L. braziliensis* in the province of Imbabura, and *L. lainsoni* in the provinces of Pichincha, Napo, Pastaza, and Morona Santiago. Amazon cases had a twice as long median health-seeking delay as Pacific cases. Prolonged health-seeking delay (≥ one month) was associated with older age, Amerindian ethnicity, infection at lower altitudes, non-ulcerative lesions, and lesions on the lower limbs.

This is the first study to report the causative *Leishmania* species in over 130 Ecuadorian CL patients, including a representative group from the Amazon region [10]. Former studies on a limited number of CL samples from the northern Ecuadorian Amazon provinces Sucumbíos and Orellana reported the mixed presence of *L. guyanensis*, *L. braziliensis*, and *L. lainsoni* whilst suggesting that *L. braziliensis* was the only causing species in the south [14, 15]. Our study, however, discovered that non-*L. braziliensis* species cause lesions in approximately half of the CL patients infected in the southern Amazon provinces. In Ecuador, *L. braziliensis* is the only species associated with a high risk of ML [45, 46]. To further identify high-risk parishes, cantons, or regions (e.g. depending on the altitude), the number of patients with species determination is insufficient. Therefore, we recommend a study on *Leishmania* species distribution including more Amazon-infected CL patients.

We report only a couple of *L. braziliensis-*caused CL cases from the Pacific region and do not confirm an increase as suggested by Kato et al. In the Pacific region, the health-seeking delay was relatively short, allowing rather prompt initiation of treatment and a subsequent decrease in the risk of ML [47]. Thus, a sustained low *L. braziliensis* prevalence combined with prompt initiation of CL treatment possibly explains why ML is rare in the Pacific region and we do not recommend routine follow-up of CL cases for ML [45, 48].

*L. braziliensis* was indistinguishable from *L. peruviana* during the comparison of a Cyt B gene fragment with the NCBI database and in the obtained phylogenetic tree. This has been reported before in Peruvian samples, but not in Ecuador [29]. To differentiate *L. braziliensis* from *L. peruviana*, we used a single nucleotide polymorphism of the MPI gene as described by Tsukayama et al. The reliability of Tsukayama’s method has been questioned as the strains might lack diversity and some species diagnostic polymorphisms might be present in other species [30, 49]. None of the MPI sequences obtained in this study contained the *L. peruviana*-specific allele at the position described by Tsukayama et al. and therefore we suppose that *L. braziliensis* was indeed the infecting species [30]. Nevertheless, for future species determination studies in Ecuador, we recommend the use of heat shock protein 70 gene fragment sequencing because it avoids an additional PCR and sequencing step [50, 51]. The phylogenetic tree divided *L. guyanensis* samples into a Pacific and Amazon predominant subclade. This suggests that a region-specific mutational development has taken place and is in agreement with Calvopiña et al. who reported region-specific zymodeme variations in Ecuador that seemed to be associated with the clinical presentation of patients [52]. Such variations underscore the importance of a regional analysis of CL case presentation, as done in this paper, as well as diagnostic test accuracy. Follow-up of patients in a prospective study would disclose the development of treatment resistance, illness recurrence, and the risk of ML, which cannot be assessed in this cross-sectional investigation.

Five *L. braziliensis* samples showed at least one “A” allele at location 1082 of the MPI gene [30]. This mutation has not been described before and seems to be Ecuadorian. Genetically complex *Leishmania* strains have been described in Ecuador before and show the importance of continuously validating and updating species determination methods [53].

This study compares a low (Pacific) with a high (Amazon) *L. braziliensis* endemic area. Younger age and a higher percentage of lesions on the head of patients infected in the Pacific are in agreement with former studies [54, 55]. This might be explained by different reservoirs and transmitting vectors in the Pacific region compared to the Amazon, but evidence on both is absent. The current hypothesis is that *Leishmania* transmission is peri-domestic in the Pacific region and occupational (agriculture, military, and hunting amongst others) in the Amazon [10, 15, 25]. The vectors, who tend to fly low to the ground, might bite children at younger ages during peri-domestic transmission in the Pacific region with a higher risk of bites on the head [56, 57].

A longer health-seeking delay for Amazon CL patients is a new finding though not unexpected as the geographical distances and physical barriers to travel to health centers in the Amazon are higher compared to the Pacific region. In the Amazon, the road network starts in the highlands and descends into the rainforest lowlands until 700 to 250m above sea level in the provinces that included patients for this study. This results in limited access to health care in the lowlands and might be an explanation for prolonged health-seeking delay [17]. Older age, Amerindian ethnicity, infection at lower altitudes, and lesions more often on the lower limbs (that are easier to cover than lesions on the head) are significantly associated with the Amazon region and may all contribute to the prolonged health-seeking delay in the Amazon. These findings might be triggered by the stigma expressions towards Amazon CL patients that were found by our team during qualitative interviews and observations that are being published separately [58, 59]. Other determinants of health-seeking delay are occupation, educational level, income, marital status, time to the nearest health center, visit to a traditional healer, and possible doctors delay. They may influence health-seeking delay, but do not appear to be related to the longer health-seeking delay in the Amazon, as adding ‘infected in the Amazon’ did not improve the binary multivariable model [60]. Non-ulcerative lesions are not associated with the Amazon region and the association with prolonged health-seeking delay might be explained by a decreased recognition by the patient population as well as by health professionals which should be clarified in a future study. Health-seeking delay was self-reported in this study, which could have resulted in under- or overestimations. We recommend a future study on CL health-seeking delay in the Amazon that includes more determinants and applies methods (e.g. relating to life events) to improve reliability [61].

Former studies have shown that parasite load is inversely related to the duration of the lesion [62–64]. Lesion parasite paucity lowers the possibility of finding amastigotes in smear slides (currently the gold standard in Ecuador). Therefore, the longer health-seeking delay in the Amazon might influence CL test accuracy and we recommend the evaluation of smear slide microscopy in the Amazon. In addition to the lesion type, number, and body location of skin lesions, this study might have included the diameter, the aspect (wet or dry), and smell which could have differed per region and or impacted health-seeking delay.

As this study included patients without restrictions from both private and public health centers from main Ecuadorian CL clusters providing free CL treatment, we consider that the results may be generalizable for the CL patient population of included areas and in a lower degree for the entire country.

## Conclusion

Our study on 245 confirmed Ecuadorian CL patients, including the causative species determination in 135 samples, shows a sustained low prevalence of *L. braziliensis* in the Pacific region. Additionally, it discovers the presence of *L. guyanensis* in the Napo, Pastaza, and Morona Santiago provinces, *L. braziliensis* in the Imbabura province, and *L. lainsoni* in the Pichincha, Napo, Pastaza, and Morona Santiago provinces. The longer health-seeking delay and a genetically different *L. guyanensis* subclade in the Amazon compared to the Pacific region reveal the need for region-specific analysis of CL test accuracy. Prolonged health-seeking delay was associated with older age, Amerindian ethnicity, infection at lower altitudes, non-ulcerative lesions, and lesions on the lower limbs which possibly result from limited access to health care and stigma. We recommend larger studies on the distribution of *Leishmania* species in Amazon CL cases and regional studies of determinants of health-seeking delay.

## Electronic supplementary material

Below is the link to the electronic supplementary material.

Additional file 1. Provincial distribution of infecting Leishmania species in 135 patients with cutaneous lesions from the Ecuadorian subtropical Pacific and Amazon regions.

Additional file 2. Individual patient variables and laboratory results.

Additional file 3. Frequencies of the genotypes identified at position 1082 in the MPI gene.

## Data Availability

All data generated or analyzed during this study are included in this published article and its supplementary information files. The cytochrome B sequences were submitted to GenBank with accession numbers: OQ608467-OQ608601. The MPI sequences were submitted to GenBank with accession numbers: OQ608603-OQ608620 [32].

## List of abbreviations

CL: Cutaneous Leishmaniasis
Cyt B: Cytochrome B
hTNF: Human Tumor Necrosis Factor
ML: Mucosal lesions
MPI: Mannose Phosphate Isomerase
qPCR: Real-time PCR
rDNA: Ribosomal DNA

## References

[1] de Vries HJ, Schallig HD, Cutaneous Leishmaniasis. A 2022 updated narrative review into diagnosis and management developments. Am J Clin Dermatol. 2022:1–18. 10.1007/s40257-022-00726-8 PMC9472198 36103050

[2] Saeedi M, Leishmaniasis Geneva, WHO. 2022. https://www.who.int/news-room/fact-sheets/detail/leishmaniasis. Accessed: 25 January 2022.

[3] Hotez PJ, Pecoul B, Rijal S, Boehme C, Aksoy S, Malecela M (2016). Eliminating the neglected tropical diseases: translational science and new technologies. PLoS Negl Trop Dis.

[4] Hotez PJ, Aksoy S, Brindley PJ, Kamhawi S. What constitutes a neglected tropical disease? Public Library of Science San Francisco, CA USA; 2020. p. e0008001. 10.1371/journal.pntd.0008001 PMC6991948 31999732

[5] Organización Panamericana de la Salud. Leishmaniasis 2022 https://www.paho.org/es/temas/leishmaniasis. Accessed: 22 December 2022.

[6] Alemayehu B, Alemayehu M (2017). Leishmaniasis: a review on parasite, vector and reservoir host. Health Sci J.

[7] Alvar J, Vélez ID, Bern C, Herrero M, Desjeux P, Cano J (2012). Leishmaniasis worldwide and global estimates of its incidence. PLoS ONE.

[8] Henríquez-Trujillo AR, Coral-Almeida M, Hinojosa MC. The burden of cutaneous and mucocutaneous leishmaniasis in Ecuador (2014–2018), a national registry-based study. bioRxiv. 2019:751446.

[9] Urutseg. Ecuador physical map 2014 https://en.m.wikipedia.org/wiki/File:Ecuador_relief_location_map.svg. Accessed: 20 January 2023.

[10] Hashiguchi Y, Velez LN, Villegas NV, Mimori T, Gomez EAL, Kato H (2017). Leishmaniases in Ecuador: Comprehensive review and current status. Acta Trop.

[11] Encalada E, Población. Superficie (KM2), Densidad Poblacional a nivel Parroquial Quito: INEC; 2010 https://www.ecuadorencifras.gob.ec/wp-content/plugins/download-monitor/download.php?id=311&force=1. Accessed: 08 June 2022.

[12] INEC. Proyección de la Poblacion Ecuatoriana, por Años Calendario, Según Regiones, Provincias y Sexo Quito: INEC. 2010. https://www.ecuadorencifras.gob.ec//wp-content/descargas/Boletines/Proyecciones_poblacionales_cantonales/PROYECCION+PROVINCIAS,+SEXOS+Y+AREAS+2010+-+2020.xlsx. Accessed: 08 June 2022.

[13] INEC. Proyección de la Poblacion Ecuatoriana, por Años Calendario, Según Cantones Quito: INEC. 2010. https://www.ecuadorencifras.gob.ec/documentos/web-inec/Poblacion_y_Demografia/Proyecciones_Poblacionales/proyeccion_cantonal_total_2010-2020.xlsx. Accessed: 08 June 2022.

[14] Kato H, Gomez EA, Martini-Robles L, Muzzio J, Velez L, Calvopiña M (2016). Geographic distribution of Leishmania Species in Ecuador based on the cytochrome B gene sequence analysis. PLoS Negl Trop Dis.

[15] Calvopina M, Armijos RX, Hashiguchi Y (2004). Epidemiology of leishmaniasis in Ecuador: current status of knowledge -- a review. Mem Inst Oswaldo Cruz.

[16] Lopez G. Población que se Autoidentificó Indigena, Segú Provincia de Empadronamiento, Nacionalidad o Pueblo Indigena al que Pertenece y Sexo Quito: INEC; 2010 https://www.google.com/url?sa=t&rct=j&q=&esrc=s&source=web&cd=&ved=2ahUKEwi6per4kJv2AhVCNewKHUBlBewQFnoECBMQAQ&url=https%3A%2F%2Fwww.ecuadorencifras.gob.ec%2Fwp-content%2Fplugins%2Fdownload-monitor%2Fdownload.php%3Fid%3D339%26force%3D0&usg=AOvVaw1sZMlqB_kh_8O316Yy4T8I. Accessed: 25 February 2022.

[17] Benabent Fernández de Córdoba M. El transporte público terrestre y la accesibilidad, instrumentos para el análisis funcional del sistema de asentamientos: el caso de Ecuador. Volume 6. Estoa Revista de la Facultad de Arquitectura y Urbanismo de la Universidad de Cuenca; 2017. pp. 99–122. 11.

[18] Villacis B, Carrillo D. País atrevido: la nueva cara sociodemográfica del Ecuador. Quito: Instituto Nacional de Estadística y Censos (INEC); 2012. Available from: https://www.ecuadorencifras.gob.ec/wp-content/descargas/Libros/Economia/Nuevacarademograficadeecuador.pdf.

[19] Rueda Miranda TM. Etnia y discriminación laboral; caso indígena en el Ecuador período 2010–2018: Riobamba. Universidad Nacional de Chimborazo; 2021.

[20] Siguencia Ibadango SK (2018). Discriminación salarial por etnia en el período 2010–2017 en el.

[21] CONFENIAE, Nacionalidades Puyo CONFENIAE. 2022. https://confeniae.net/nacionalidades. Accessed: 24 February 2022.

[22] Romero GA, de Farias Guerra MV, Paes MG, Macêdo VdO (2001). Comparison of cutaneous leishmaniasis due to Leishmania (Viannia) braziliensis and L.(V.) guyanensis in Brazil: clinical findings and diagnostic approach. Clin Infect Dis.

[23] Aronson NE, Joya CA (2019). Cutaneous leishmaniasis: updates in diagnosis and management. Infect Dis Clin N Am.

[24] Espin CJT, Procel MC (2021). Leishmaniasis en el Ecuador: revisión bibliográfica. Mediciencias UTA.

[25] Conteron E (2015). Prevalencia de leishmaniasis en el area II de Pastaza, asociados a factores de riesgo que influyen en el desarrollo de la enfermedad.

[26] Ministerio De Salud Pública (2013). Manual de procedimientos del subsistema alerta acción SIVE – ALERTA.

[27] Bastidas CA, Villacrés-Granda I, Navarrete D, Monsalve M, Coral-Almeida M, Cifuentes SG (2019). Antibiotic susceptibility profile and prevalence of mecA and lukS-PV/lukF-PV genes in Staphylococcus aureus isolated from nasal and pharyngeal sources of medical students in Ecuador. Infect Drug Resist.

[28] Bezemer JM, Merckx J, Freire Paspuel BP, Calvopiña M, de Vries HJC, Schallig HDFH et al. Diagnostic accuracy of qPCR and microscopy for cutaneous leishmaniasis in rural Ecuador: a bayesian latent class analysis. 2023. 10.1371/journal.pntd.0011745 PMC10686511 38019756

[29] Kato H, Cáceres AG, Hashiguchi Y (2016). First evidence of a hybrid of Leishmania (Viannia) braziliensis/L.(V.) peruviana DNA detected from the phlebotomine sand fly Lutzomyia tejadai in Peru. PLoS Negl Trop Dis.

[30] Tsukayama P, Lucas C, Bacon DJ (2009). Typing of four genetic loci discriminates among closely related species of New World Leishmania. Int J Parasitol.

[31] National Library of Medicine. Basic Local Alignment Search Tool. 2022 https://blast.ncbi.nlm.nih.gov/Blast.cgi. Accessed: 02 December 2022.

[32] National Library of Medicine. GenBank. 2023 https://www.ncbi.nlm.nih.gov/genbank/. Accessed: 10 March 2023.

[33] Carrera F, Alejandro D (2019). Identificación y diferenciación molecular por la técnica de la Reacción de la Cadena Polimerasa (PCR) múltiple de subgéneros de Leishmania (Viannia y Leishmania) en el Ecuador.

[34] CODENPE (2011). Modulo 3: Interculturalidad.

[35] DGAC. Internet Flight Information Service. 2022 http://www.ais.aviacioncivil.gob.ec/. Accessed: 02 December 2022.

[36] topographic-map.com. Mapa topográfico Ecuador. 2022 https://es-ec.topographic-map.com/. Accessed: 02 December 2022.

[37] CASTOR. CASTOREDC. 2022 https://www.castoredc.com/. Accessed: 02 December 2022.

[38] IBM (2021). SPSS Statistics for Windows. Version 28.0. Armonk.

[39] Mackenzie CS, Heath PJ, Vogel DL, Chekay R (2019). Age differences in public stigma, self-stigma, and attitudes toward seeking help: a moderated mediation model. J Clin Psychol.

[40] Paz-Soldan VA, Alban RE, Dimos Jones C, Powell AR, Oberhelman RA (2014). Patient reported delays in seeking treatment for tuberculosis among adult and pediatric TB patients and TB patients co-infected with HIV in Lima, Peru: a qualitative study. Front Public Health.

[41] Alcalde-Rubio L, Hernández-Aguado I, Parker LA, Bueno-Vergara E, Chilet-Rosell E (2020). Gender disparities in clinical practice: are there any solutions? Scoping review of interventions to overcome or reduce gender bias in clinical practice. Int J Equity Health.

[42] Bautista-Valarezo E, Duque V, Verhoeven V, Mejia Chicaiza J, Hendrickx K, Maldonado-Rengel R (2021). Perceptions of ecuadorian indigenous healers on their relationship with the formal health care system: barriers and opportunities. BMC Complement Med Ther.

[43] Hu R, Ramdas S, Nieuwkerk P, Reis R, Lai AFRFM, de Vries HJC (2020). Body location of “New World” cutaneous leishmaniasis lesions and its impact on the quality of life of patients in Suriname. PLoS Negl Trop Dis.

[44] Von Elm E, Altman DG, Egger M, Pocock SJ, Gøtzsche PC, Vandenbroucke JP (2007). The strengthening the reporting of Observational Studies in Epidemiology (STROBE) statement: guidelines for reporting observational studies. Ann Intern Med.

[45] Ronquillo TEF, Almeida FRT (2012). Reporte de lesiones mucosas en leishmaniosis tegumentaria americana, en el litoral (Costa). Ecuatoriano Rev patol trop.

[46] Calvopina M, Guevara AG, Armijos RX, Hashiguchi Y, Davidson RN, Cooper PJ (2004). Itraconazole in the treatment of New World mucocutaneous leishmaniasis. Int J Dermatol.

[47] Machado-Coelho GL, Caiaffa WT, Genaro O, Magalhaes PA, Mayrink W (2005). Risk factors for mucosal manifestation of american cutaneous leishmaniasis. Trans R Soc Trop Med Hyg.

[48] Ministerio de Salud Publica (2022). Subsistema de Vigilancia SIVE-ALERTA Enfermedades Transmitidas por Vectores Ecuador 2021.

[49] Schönian G, Kuhls K, Mauricio I (2011). Molecular approaches for a better understanding of the epidemiology and population genetics of Leishmania. Parasitology.

[50] Van der Auwera G, Dujardin J-C (2015). Species typing in dermal leishmaniasis. Clin Microbiol Rev.

[51] Montalvo A, Fraga J, Maes I, Dujardin J-C, Van der Auwera G (2012). Three new sensitive and specific heat-shock protein 70 PCRs for global Leishmania species identification. Eur J Clin Microbiol Infect Dis.

[52] Calvopina M, Armijos RX, Marco JD, Uezato H, Kato H, Gomez EA (2006). Leishmania isoenzyme polymorphisms in Ecuador: relationships with geographic distribution and clinical presentation. BMC Infect Dis.

[53] Kato H, Cáceres AG, Gomez EA, Tabbabi A, Mizushima D, Yamamoto DS (2021). Prevalence of genetically complex leishmania strains with hybrid and Mito-Nuclear discordance. Front Cell Infect Microbiol.

[54] Armijos R, Weigel M, Izurieta R, Racines J, Zurita C, Herrera W (1997). The epidemiology of cutaneous leishmaniasis in subtropical Ecuador. Tropical Med Int Health.

[55] Amunárriz M. Estudios sobre patologías tropicales en la Amazonia ecuatoriana: Cicame; 1991.

[56] Faiman R, Kirstein O, Moncaz A, Guetta H, Warburg A (2011). Studies on the flight patterns of foraging sand flies. Acta Trop.

[57] Gaglio G, Brianti E, Napoli E, Falsone L, Dantas-Torres F, Tarallo VD (2014). Effect of night time-intervals, height of traps and lunar phases on sand fly collection in a highly endemic area for canine leishmaniasis. Acta Trop.

[58] Vargas V, Bezemer J, Calvopina M, Schallig H, Ortega F, Salazar N et al. Perceptions of American tegumentary leishmaniasis in Ecuador: questioning the visuality of aesthetic stigma. 2022.

[59] Bezemer JM, Calvopiña Hinojosa M, Corral Zabala AE, Ortega Pérez F, Vargas Román VC, Schallig HDFH (2022). Quality of life of cutaneous leishmaniasis suspected patients in the ecuadorian Pacific and Amazon regions: a cross sectional study. BMC Infect Dis.

[60] Henry M, GalAn N, Teasdale K, Prado R, Amar H, Rays MS (2016). Factors contributing to the delay in diagnosis and continued transmission of leprosy in Brazil–an explorative, quantitative, questionnaire based study. PLoS Negl Trop Dis.

[61] Glasner T, Van der Vaart W (2009). Applications of calendar instruments in social surveys: a review. Qual Quantity.

[62] Jara M, Adaui V, Valencia BM, Martinez D, Alba M, Castrillon C (2013). Real-time PCR assay for detection and quantification of Leishmania (Viannia) organisms in skin and mucosal lesions: exploratory study of parasite load and clinical parameters. J Clin Microbiol.

[63] Pereira LOR, Moreira RB, de Oliveira MP, Reis SO, de Oliveira Neto MP, Pirmez C (2017). Is Leishmania (Viannia) braziliensis parasite load associated with disease pathogenesis?. Int J Infect diseases IJID: official publication Int Soc Infect Dis.

[64] Chico ME, Guderian RH, Cooper PJ, Armijos R, Grogl M (1995). Evaluation of a direct immunofluorescent antibody (DIFMA) test using Leishmania genus-specific monoclonal antibody in the routine diagnosis of cutaneous leishmaniasis. Rev Soc Bras Med Trop.

