# Supplementary material for: Leishmania species and clinical characteristics of Pacific and Amazon cutaneous leishmaniasis in Ecuador and determinants of health-seeking delay: a cross-sectional study
Source: BMC Infect Dis. 2023 Jun 12;23:395. doi: 10.1186/s12879-023-08377-8 (PMC10258766; doi:10.1186/s12879-023-08377-8)
Supplement: Supplementary file 1 — Additional file 1. Provincial distribution of infecting Leishmania species in 135 patients with cutaneous lesions from the Ecuadorian subtropical Pacific and Amazon regions. [file 12879_2023_8377_MOESM1_ESM.docx]

**Provincial distribution of infecting *Leishmania* species in 135 patients with cutaneous lesions from the Ecuadorian subtropical Pacific and Amazon regions. Total (%)**

|  | ***L. guyanensis*** | ***L. braziliensis*** | ***L. lainsoni*** | **Total** |
| --- | --- | --- | --- | --- |
| **Pacific provinces** | **83 (93)** | **5 (6)** | **1 (1)** | **89 (100)** |
| Pichincha | 78 (94) | 4 (5) | 1 (1) | 83 (100) |
| Imbabura | 1 (50) | 1 (50) | 0 (0) | 2 (100) |
| Santo Domingo | 2 (100) | 0 (0) | 0 (0) | 2 (100) |
| Manabi | 1 (100) | 0 (0) | 0 (0) | 1 (100) |
| Guayas | 1 (100) | 0 (0) | 0 (0) | 1 (100) |
| **Amazon Provinces** | **19 (41)** | **21 (46)** | **6 (13)** | **46 (100)** |
| Sucumbios | 2 (100) | 0 (0) | 0 (0) | 2 (100) |
| Napo | 1 (25) | 0 (0) | 3 (75) | 4 (100) |
| Orellana | 1 (50) | 1 (50) | 0 (0) | 2 (100) |
| Pastaza | 13 (39) | 18 (55) | 2 (6) | 33 (100) |
| Morona Santiago | 2 (40) | 2 (40) | 1 (20) | 5 (100) |
| **Total** | **102 (76)** | **26 (19)** | **7 (5)** | **135 (100)** |
