# Supplementary material for: Leishmania species and clinical characteristics of Pacific and Amazon cutaneous leishmaniasis in Ecuador and determinants of health-seeking delay: a cross-sectional study
Source: BMC Infect Dis. 2023 Jun 12;23:395. doi: 10.1186/s12879-023-08377-8 (PMC10258766; doi:10.1186/s12879-023-08377-8)
Supplement: Supplementary file 3 — Additional file 3. Frequencies of the genotypes identified at position 1082 in the MPI gene. [file 12879_2023_8377_MOESM3_ESM.docx]

**Frequencies of the genotypes identified at position 1082 in the MPI gene [1].**

| **Genotype^a^** | **N (%)** |
| --- | --- |
| **GA** | 0 (0) |
| **GT** | 0 (0) |
| **GG** | 0 (0) |
| **GC** | 0 (0) |
| **CC** | 13 (72) |
| **CA** | 4 (22) |
| **AA** | 1 (6) |
| **Total** | 18 (100) |

^a^The G allele is supposed to be specific for *L. peruviana*

1. Tsukayama P, Lucas C, Bacon DJ. Typing of four genetic loci discriminates among closely related species of New World Leishmania. International journal for parasitology. 2009;39(3):355-62.
